# Factors associated with the length of delay with tuberculosis diagnosis and treatment among adult tuberculosis patients attending at public health facilities in Gondar town, Northwest, Ethiopia

**DOI:** 10.1186/s12879-017-2240-0

**Published:** 2017-02-14

**Authors:** Selamsew Bogale, Ermias Diro, Atsede Mazengia Shiferaw, Melaku Kindie Yenit

**Affiliations:** 1 Department of Internal medicine, University of Gondar, Gondar, Ethiopia; 2 Department of Health Informatics, University of Gondar, Gondar, Ethiopia; 3 Department of Epidemiology and Biostatistics, University of Gondar, 196, Gondar, Ethiopia

**Keywords:** Delay, TB diagnosis and treatment, Northwest Ethiopia, 2016

## Abstract

**Background:**

Early diagnosis and prompt treatment is essential for an effective tuberculosis (TB) control program. However, significant proportion of cases remains undiagnosed and untreated. Delay in diagnosis and treatment increases transmission. Hence, the study assessed the length of delay and associated factors with tuberculosis diagnosis and treatment among adults attending public health facilities in Gondar town, Northwest Ethiopia.

**Method:**

An institution based cross-sectional study was conducted from February to May, 2016. A total of 296 adults who came to health facilities for treatment for pulmonary TB from February to May, 2016, were included in the study. Data were collected using a structured questionnaire through interviewing and record review, cleaned, coded, and entered into Epi-info version 3.5.3, and transferred into SPSS version 20.0 for further statistical analysis. A *p*-value of less than 0.05 at multiple linear regression analysis was considered statistically significant.

**Result:**

The mean duration of the total delay (in days) for tuberculosis diagnosis and initiation of treatment was 41.6 days (SD = 16.6). In this study, the mean duration of patient delay and the median health system delay were 33.9 days (SD = 14) and 5 days (IQR = 4–7), respectively. Total delay for TB diagnosis and treatment was shorter among HIV positive people (β:-12.62, 95% CI: −20.72,-4.53). Longer patient delay was noted among rural dwellers (β: 8.0, 95% CI: 5.26, 10.75); increased household income (β:-0.006, 95% CI: −0.008,-0.004) was associated with a shorter delay. Health system delay was positively associated with seeking care from more than one health care providers (β: 0.28, 95% CI: 0.23, 0.34) and seeking initial care from primary level health care facilities (β: 0.10, 95% CI: 0.07, 0.13).

**Conclusion:**

In this study, the majority of patients faced delayed in seeking health care and continued as sources of infection. Longer days of delay for TB diagnosis and treatment were noted among rural residents, who seek health care from informal care providers, and receive initial care from primary level health care facilities. In contrast, the length of delay for TB diagnosis and treatment was shorter among HIV positive people and individuals with increased household income. Therefore, public awareness on the symptoms of tuberculosis and seeking health care early is essential. Moreover, early diagnosis and treatment, especially among the rural dwellers and the poor should be focused.

## Background

Tuberculosis (TB) remains a major public health problem throughout the world [1, 2]. About one-third of the world’s population is estimated to be infected with the bacilli and become at risk of developing active TB infection [2]. In 2014, about 9.6 million new TB cases and over 1.5 million deaths from the diseases were reported; 95% of these deaths occurred in low and middle income countries. Africa is the region with the most burden of TB cases with an estimated prevalence of 281 all forms of TB cases per 100,000 inhabitants which is doubled of the global average of 133 cases per 100,000 [3, 4]. According to the global tuberculosis report most countries shows progress in reducing the burden of tuberculosis though it is not as targeted by the millennium development goal. Considering this unmet target and to transform the world, the international community develops a new agenda of ending TB epidemic by 2030 in the newly adopted Sustainable Development Goals [5].

Effective TB control program through early diagnosis and treatment is an essential strategy to decrease the burden of the disease [2, 6]. Most of the risk of TB infection occurs between the contacts of the infectious cases and before the initiation of treatment [7–10]. Early screening of presumptive TB cases starting at the community, providing rapid diagnosis, and treating the cases early at health facilities reduces the risk of disease transmission as a result the reduction of time between the onset of symptoms and the initiation of treatment [2, 11]. Thus individuals who had cough for two weeks or more are requested to go to health facilities for diagnosis and early treatment [12]. As one of the major tools to diagnose tuberculosis, direct microscopy is not only cost effective but also simple and capable of producing reliable result, within two consecutive days [13–15]. The National (Ethiopia) TB Control Program recommends collecting three sputum specimens for pulmonary TB (PTB), and that individual who have at least two smear positive results or a single smear positive result supported by radiographic abnormalities be classified as pulmonary smear positive (PTB^+^). While those with three initial smears negative examinations but showed major symptoms of TB are confirmed by gene-xpert or culture [16, 17]. However this, low case detection rate (CDR) in most nations resulting from patients’ inability to seek health care at the onset of symptoms and the delay of diagnosis and treatment remain a challenge in TB control programs [3, 13, 18, 19].

Delays in seeking health care and in providing early diagnosis and treatment increases the risk of disease transmission, and subsequently leads to death. TB diagnosis and treatment are delayed when patients wait until long after the onset of symptom to seek care (patient delay), or when care providers take too long to diagnose and treat the patients who sought care (health system delay) [14]. The length of delay was significantly longer among low and middle income countries than in the developed nations. The longest total delay in TB diagnosis and treatment was noted in Afghanistan (356.0 days) [20]. In most studies conducted elsewhere, it was noted that patient delay was longer than health system delay [20–22]. A cross-sectional study among smear positive pulmonary TB patients in Afghanistan reported that patient delay (205 days) was longer than health system delay (151 days). In Nigeria, the median total delay, patient delay, and health system delay was 11, 8, and 3 weeks, respectively [21]. In Angola, the median total time that elapsed from the onset of symptoms to diagnosis was 45 days,while the median patient delay and health system delay was 30 and 7 days, respectively [22]. A cross sectional study in Chad reported that the median patient delay, system delay, and total delay were 15, 36, and 57.5 days, respectively [23]. In Ethiopia, though there are regional variations in total delay in TB diagnosis and treatment, it generally ranged from 40–97 days [8, 24–28]. As demonstrated by various reports, delay in diagnosis and treatment is found to be affected by various factors, but it is mainly related to patients’ health care seeking behavior and health system provision of prompt diagnosis and treatment [22, 25, 29, 30]. Among the various factors reported, residence [22, 26], type of health facility [22], and seeking care from other than health care providers [29–31] were associated with the delay for TB diagnosis and treatment.

Ethiopia ranks 10th among 22 high TB burden countries (HBCs) and the disease remains one of the leading causes of mortality and morbidity. According to the 2014 WHO report, the incidence and prevalence of all forms of TB cases were 224 and 211 per 100,000 populations, respectively. Though there has been some achievement in the process of reducing the incidence of tuberculosis as targeted in the Millennium Development Goal (MDG),, still one-third of the suspected cases are not detected and continue a source of TB infection [32, 33]. Therefore, to determine the length of delay in seeking health care, early diagnosis and initiation of treatment can improve the detection rate and will have further role in the success of TB control programs.

## Methods

### Study setting and design

An institution-based cross-sectional study was conducted among pulmonary TB patients, who started TB treatment from February to May, 2016. The study was conducted at public health facilities of Gondar town. The town has eight health centers and one referral hospital providing sputum smear examination and direct observed short course on TB treatment (DOTS) (Gondar Town Adminstration Health Office: Annual Report of Gondar Town Administration Health Office, unpublished).

### Study participants, sample size and sampling procedure

All bacteriologically confirmed pulmonary TB cases greater than 18 years of age who were on treatment at an intensive phase were the population under study. The sample size was calculated using the single population mean formula and by considering the following assumptions: 44 days mean time for total delay [34], a 95% level of confidence, 5% margin of error, and 5% non-response rate. Thus, a minimum sample size of 311 was obtained. As a result, all eligible TB patients attending the selected public health facilities during the study period were included consecutively. However, to avoid over-counting, a reminder note was attached to the transfer sheet when a patient was transferred from one health facility to another.

### Data collection tools and procedure

Data were collected using a pretested structured questionnaire which was developed from the WHO multi-country study designed to estimate the length of delay of TB treatment [14, 35]. To maintain consistency, the questionnaire was first translated from English to Amharic (the native language of the study area) and retranslated to English by professional translators and public health experts. Data were collected by interviewing and record review. Nine data collectors and two supervisors were selected for the study. Two days’ intensive training regarding the objective of the study, and confidentiality of information was given to data collectors and supervisors. Awareness was created regarding the objective of the study, and respondents were made aware that their responses would not affect the possible treatments they needed.

### Operational definitions

**Fig. 1:**
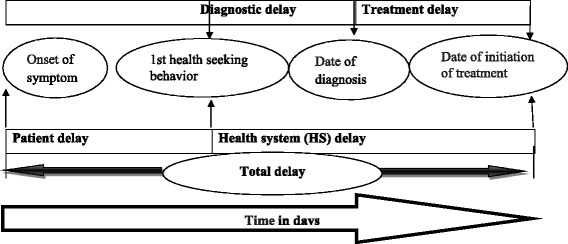
Flow-chart showing different delay durations contributing to total delay (Source: WHO EMRO, Diagnostic and treatment delay in TB, 2006)

Total treatment delays (TTD), the dependent variable was assessed according to the key dimensions of delays for TB diagnosis and treatment stated by WHO. The total delay for TB diagnosis and treatment was the sum of patient delay and health system delay. It was specifically defined as the time interval between the onset of a cough to the first visit to a physician or health center (patient delay), plus the health system delay, which is the interval between the first visit to the initiation of anti-TB treatment (Fig. 1) [14].

A bacteriologically confirmed TB case was referred to as a patient whose biological specimen is positive by smear microscopy or WRD (such as Xpert MTB/RIF) [36].

### Data processing and analysis

Data were entered into Epi-info version 3.5.3 and exported to a statistical package for social sciences (SPSS) version 20 for further analysis. Data cleaning was done by running frequencies. Descriptive statistics, including frequencies and proportions were computed to summarize the variables. Linear regression model was used in the process. Variables with a *p*-value of less than 0.2 in the simple linear regression analysis were entered into the multiple linear regression analysis. A *p*-value of less than 0.05 at the multiple linear regression analysis was considered statistically significant.

## Results

### Socio-demographic characteristics of TB patients

A total of two hundred ninety-six adult TB patients were included in the study. The mean age (±SD) of participants was 31.7 years (±10.1). Of the total study participants, 50.7% were females, 69.3% were urban dwellers, and 29.1% were illiterate. Most of the respondents (88.5%) were Orthodox Christians and the rest (9.8% and 1.7%, respectively) were Muslims and other religions. Participants who had access to health facilities in 30 min of walking time were 61.8%. More than half of the respondents had a monthly household income ranging between ETB 501–1000 [Table 1].

**Table 1:** Socio-demographic characteristics of TB patients attending at public health facilities in Gondar town, Northwest Ethiopia, 2016

Variables	Frequency	Percent
Sex		
Male	146	49.3
Female	150	50.7
Age category (year)		
18–24	71	24
25–29	69	23.3
30–34	51	17.2
35–39	50	16.9
40–44	33	11.1
45–49	6	2.0
≥50	71	24
Religion		
Orthodox	262	88.5
Muslims	29	9.8
Others^a^	5	1.7
Ethnicity		
Amhara	255	86.1
Kimant	29	9.8
Others^b^	12	4.1
Residence		
Urban	205	69.3
Rural	91	30.7
Marital Status		
Single	125	42.2
Married	121	40.9
Divorced	41	13.9
Others^c^	9	3.4
Educational status		
No education	86	29.1
Read and write only	58	19.6
Primary School	80	27
Secondary School	48	16.2
College/University	24	8.1
Occupation		
Un-employed	40	13.5
Self employed	84	28.4
Government employed	36	12.2
Private employed	17	5.7
Farmer	28	9.5
Student	28	9.5
Housewife	63	21.3
Household Income (ETB)		
<500 Birr	68	23
501–1000 Birr	157	53
>1000	71	24
Time to reach health facility		
<30 min	183	61.8
30–60 min	58	19.6
>60	55	18.6

^a^Protestant, ^b^Tigre, Oromo, ^c^Widowed, Separated

### Clinical and Behavioral Characteristics of respondents

About one-fifth (20.3%) of the respondents had history of exposure to tuberculosis. Among the most common symptoms reported, a significant proportion of respondents (88.9%, 72.6%, and 72.6%, respectively) had cough, fever and night sweat. More than one-third (33.1%) did not consult health care providers as the first action during their symptoms. Most of the respondents perceived that the symptoms will go away on their own, and this was one of the reasons for the delay in diagnosis and treatment. Among the total 296 TB patients, about 228 cases were identified by sputum smear microscopy, while the rest, 68 TB cases were ascertained by gene-xpert. Twenty-four percent of the TB cases were co-infected with HIV [Table 2].

**Table 2:** Clinical characteristics of TB patients attending at public health facilities in Gondar town, Northwest Ethiopia, 2016

Variables	Frequency	Percent
Previous exposure to TB patient		
Yes	60	20.3
No	236	79.7
HIV Status		
Positive	71	24
Negative	225	76
First action with onset of symptoms		
Consult Health Care Provider	139	47
Practiced self-medication	55	18.6
Visit traditional healers	29	9.8
Using non-prescribed medications from Pharmacies	73	24.7
Type of health facility first consultation		
Health Post	24	8.1
Health Center	126	42.6
Governmental Hospital	79	26.7
Private Clinic	57	19.3
Private hospital	10	3.4
No of Health Care Providers consulted		
≤ 2	34	24.5
3-4	23	16.5
≥ 5	82	59.0
Reasons of First Consultation		
Accessibility	148	50
Confidence in getting Cured	35	11.8
Service available anytime	42	14.2
Better Care	46	15.5
Smear Status		
Smear Positive	228	77
Smear Negative	68	23
Investigation TB diagnosis made		
Sputum smear microscopy	228	77
Genexpert	68	23

The behavioral characteristics of respondents indicated that most (99%) knew the kind of disease they had at the time of interview. One-third (36.5%) were aware that tuberculosis was not a hereditary disease, while only 10.1% knew that tuberculosis had a vaccine. More than half (54.1%) knew how to prevent TB [Table 3].

**Table 3:** comprehensive knowledge on tuberculosis among TB patients attending at public health facilities in Gondar town, Northwest, Ethiopia, 2016

Knowledge items	Frequency	Percent
Know the kind of disease they have		
Yes	293	99
Know tuberculosis is not hereditary		
Yes	108	36.5
Know tuberculosis is contagious		
Yes	290	98
Does tuberculosis curable		
Yes	283	95.6
Is tuberculosis vaccine preventable		
Yes	30	10.1
Know the duration of ant tuberculosis treatment		
Yes	253	85.5
Know TB prevention methods		
Yes	160	54.1

### Length of delay in TB Diagnosis and Initiation of treatment

In this study, the mean total days of delay for TB diagnosis and initiation of treatment were 41.6 (SD: 16.6). The minimum and maximum delay for TB diagnosis and treatment were 14 and 95 days, respectively. Longer days of delay in seeking health care were observed among patients. To be exact, the mean patient and the median health system delays were 33.9 (SD: 14) and 5 (IQR: 4, 7) days, respectively [Table 4].

**Table 4:** Types of delay of TB diagnosis and initiation of treatment among TB patients attending public health facilities of Gondar town, Northwest Ethiopia, 2016

Type of Delay		Value
Patient Delay (days)	Mean	33.9
	SD	14
	Min-Max	10–90
Health system Delay (days)		
	Median	5
	IQR	4–7
	Min-Max	3–30
Total Delay (days)		
	Mean	41.6
	SD	16.6
	Min-Max	14 – 95

**Fig. 2:**
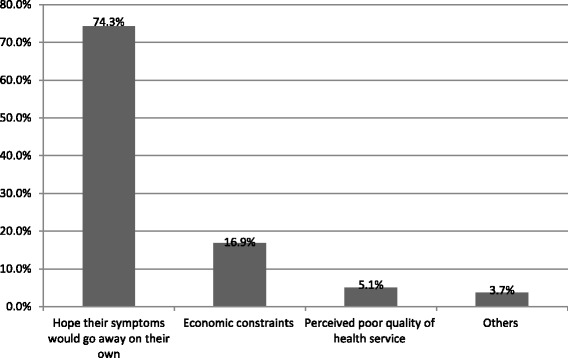
Perceived causes of delay in health seeking health care among TB patients in public health institution of Gondar town, Northwest Ethiopia, 2016

The length of total delay was similar among male and female respondents, while it was longer among rural dwellers, and those who had below ETB 500 monthly income. The major perceived causes of delay of TB diagnosis and treatment for 74.3%, 16.9%, and 5.1% of patients were reliance on mere hope that symptoms would clear up of their own accord, economic constraints, and imagined poor health care service, respectively [Fig. 2].

### Factors associated with patient delay in seeking tuberculosis diagnosis

In the multiple linear regression analysis, rural residence (β:8, 95% CI: 5.26,10.75), and seeking care from informal providers at onset of symptoms (β:8.09, 95% CI: 5.50,10.69) were factors associated with longer patient delays; while increased household income (β:0.006,95% CI: −0.008,-0.004), and HIV seropositivity (β:8.9, 95% CI: −12.02,–5.94) were associated with the shorter patient delays. This study specifically showed that patient delays among individuals who live in rural areas increased by 8 days compared with patients from urban settlements;, individuals who seek care from informal care providers at onset of symptoms had 8.1 more days to seek health care for diagnosis, compared with those who seek health care from formal health care providers. The study also indicated that for a unit increase in household income (in Birr), patient delay decreased by 0.006 days, which means when a household income increased by ETB 1000 per month, patient delay decreased by 6 days. Individuals who were HIV positive sought health care nearly 9 days earlier than HIV negative individuals [Table 5].

**Table 5:** Factors associated with patient delays among adult tuberculosis patients attending public health facilities of Gondar town, Northwest Ethiopia, 2016

Variables	Simple Linear Regression	Multiple Linear Regression
	β (95% CI)	β (95% CI)
House hold income	−0.009 (−0.011, −0.006)	−0.006 (−0.008, −0.004)
Rural residency	9.88 (6.58, 13.19)	8.00 (5.26, 10.75)
HIV positive status	−14.68 (−18.04, −11.32)	−8.97 (−12.02, −5.94)
seeking care from informal care providers^a^	11.19 (8.22, 14.16)	8.09 (5.50, 10.69)

*B* Beta coefficient

^a^Traditional medicine, non-prescribed medication from drug store

### Factors associated with delay in tuberculosis diagnosis and treatment at the health system

In the multiple linear regression analysis, longer health system delays were noted among respondents who sought care from more than one health care providers (β: 0.28, 95% CI: 0.23, 0.34) and primary level health care facilities (β: 0.10, 95% CI: 0.07, 0.13) [Table 6].

**Table 6:** Factor associated with health system delay among adult tuberculosis patients’ attending public health facilities of Gondar town, Northwest Ethiopia, 2016

Variables	Simple Linear Regression	Multiple Linear Regression
	β (95% CI)	β (95% CI)
Age	0.04 (0.01, 0.07)	–
Sex	0.06 (0.01, 0.13)	–
Residency	0.14 (0.08, 0.21)	–
HIV status	−0.08 (–0.16, −0.01)	–
Seeking care from more than one health care provider	0.30 (0.23, 0.38)	0.28 (0.23,0.34)
Seeking initial care from primary level health care facilities	0.12 (0.99,0.14)	0.10 (0.07,0.13)

*B* Beta coefficient

### Factors associated with total delay in tuberculosis Diagnosis and treatment

In the simple linear regression analysis, residence, educational status, household income, time to reach a health facility, HIV status, and seeking more than one health care provider were factors associated with total delays in tuberculosis diagnosis and treatment at a *p*-value of less than 0.2. Consequently, these variables were subjected to multiple linear regression analysis, and it was noted that a longer delay of TB diagnosis and treatment was observed among rural residents (β: 10.3, 95% CI: 2.911, 17.824), and those who seek health care from more than one health care providers (β: 7.674, 95% CI: 2.972, 12.376), while increased household income (β:-0.008, 95% CI–0.013,–0.002 :) and HIV seropositivity (β:–12.62, 95% CI:–20.72,–4.525) were positively associated with shorter delays in diagnosis and treatment. Individuals from rural areas had ten extra days for TB diagnosis and treatment compared with individuals from urban areas. Similarly, household income was significantly associated with total delays of TB diagnosis and treatment. For a unit increase in household income in Birr, total delay decreased by 0.008 days, which means that as a household income increased by ETB 1000 per month, delay of TB diagnosis and treatment decreased by 8 days, and patients who were HIV positive had 12.6 shorter days for diagnosis and treatment compared with HIV negative individuals [Table 7].

**Table 7:** Factors associated with total delay of tuberculosis diagnosis and treatment among adult tuberculosis patients in public health facilities of Gondar town, Northwest Ethiopia, 2016

Variables	Simple Linear Regression	Multiple Linear Regression
	β (95% CI)	β (95% CI)
Educational status	−3.71 (−5.13,−2.28)	–
First health care facility consulted	4.69 (3.28,6.11)	–
Time to reach to the health facility	0.25 (0.19,0.29)	–
Rural residency	13.45 (9.63,17.28)	10.3 (2.91,17.82)
House hold income	−0.009 (−0.012,−0.007)	−0.008 (−0.013,−0.002)
HIV status	−17.236 (−21.20,−13.26)	−12.62 (−20.72,−4.53)
Seeking care from more than one health care provider	7.465 (1.52,13.41)	7.674 (2.97,12.37)

*B* Beta coefficient

## Discussion

Ending TB epidemic by 2030 is among the health targets of the newly adopted Sustainable Development Goals [5]. Cognizant of this initiative, early detection and prompt treatment of cases is the main strategy to reduce disease morbidity and mortality throughout the world [11]. In this study, total delay, patient and health system delays were measured to assess the length of delay in diagnosis and treatment.

In this study, mean total days of delay of TB diagnosis and treatment was 41.6 days (SD: 16.6). Out of the total 41.6 days of delay of diagnosis and treatment, the bulk of the days (33.9) of delay were patient delays. The length of delay in the health system of diagnosis and treatment was 5 days, which was significantly shorter than the patient delay. This reflects seeking health care for TB diagnosis and treatment takes longer time when compared with length of stay in the health system. In addition, it suggests that effort to increase awareness of the community to enhance seeking care to health care.

The total days of delay of TB diagnosis and treatment in this study (41.6 days) was in line with the studies conducted in Iraq (45 days) [37], and in Arsi Zone, Ethiopia (40 days) [8]. However, in this study total delay was lower than those of in studies in different regions of Ethiopia, Bale zone (97 days) [24], Afar (70.5 days) [25], Bahirdar (60 days) [26], Tigray (90 days), and East Wollega (90 days) [27, 28], and it was also lower than the findings of studies conducted in Nepal (50 days) [38], West Africa (60 days) [9], and Tanzania (120 days) [10]. The lower delay seen in this study compared with other studies might be that the majority of the participants of this study were urban dwellers who had access to better health care facilities, and the recent implementation of the Urban Health Extension Program in the study area. This suggests that effort should be made to expand and sustain these health care facilities in achieving the sustainable development goals and universal health coverage.

The finding revealed that the mean patient delay (standard deviation) was 33.9 (SD: 14) days. This finding was consistent with those of studies conducted in Ethiopia, for example Arsi Zone (22 days) [8], and Southern Ethiopia (30 days) [39], Tanzania and Zimbabwe (28 days each) [10, 40], Sudan (27 days) [41], and Thailand (30 days) [42], but it was longer than that of study conducted at Bahir Dar (21 days) [26]. On the other hand, the median health system delay (IQR) was 5 days (4–7). This finding was consistent with a report from Yemen (4 days) [37] and Arsi Zone (6 days) [8]. However, it was longer than the report from Zimbabwe (2 days) [40]. The longer health system delay in this study might be due to the turnover of experienced staff and the efficiency of health care professionals in identifying suspected cases [1, 43, 44]. Furthermore, shortage of resources of the TB control programs which was faced in most health facilities might be the reason for longer health system delays in TB diagnosis and treatment [45, 46].

Among factors which had significant associations with patient delays, patient delays among rural residents were longer by 8 days compared with urban residents. Consistent with studies elsewhere [28, 41, 47, 48], long delays among rural residents might be the inaccessibility of health care facilities. Similarly, the length of patient delay was longer among respondents who sought health care from informal care providers, which was consistent with studies conducted in Afar [25], Arsi Zone [8], and Uzbekistan [49]. In countries like Ethiopia where there are various traditional practices and poor access to quality health care, patient sough care from informal health care provider; as a result the patients might have been given inappropriate care which led to several other visits before reaching the appropriate health facility for TB care. In contrast, shorter patient delay was noted among HIV positive individuals compared with HIV negative ones. This association was similar with those of studies in Bahir Dar, Ethiopia [26], and Mozambique [31]. This might be due to the collaborative TB/HIV activities existing in the health institution which ensures early TB screening among HIV patients and vice versa [50]. In addition, strict follow-up of health care providers for HIV positive individuals might be the reason for shorter delay in seeking health care for TB diagnosis and treatment.

A shorter patient delay was reported among individuals with increased household income, which was consistent with a finding in Sudan [41]. This might be due to the role of income in enhancing the chance of seeking health care. Consistent with studies conducted elsewhere [8, 24, 40, 51], a longer health system delay was noted among individuals who seek care from more than one health care providers. Similarly, the length of health system delay was longer among individuals who sought initial care from primary level health facilities which was similar with those of studies conducted in Nigeria [21] and different regions of Ethiopia, like Arsi Zone, Bahir Dar, and Bale zone [8, 24, 26].

As a limitation, recalling the exact date of the onset of symptoms and the date of visits to health facilities might under or overestimate the delay. However, to minimize such bias reviews of medical records, national holidays, religious days, and dates of some events were put to use. The other limitation of this study, patient related factors in the health system delay, was not assessed.

## Conclusion

The delay of tuberculosis diagnosis and treatment in this study was longer than the national recommendation of two weeks (14 days). Seeking health care for diagnosis was responsible for longer delays. Longer delay of TB diagnosis and treatment were noted among rural residents, patients who sought health care from informal care providers, and those who received initial care from primary level health care facilities. In contrast, the delay of TB diagnosis and treatment was shorter among HIV positive people and individuals with increased household income. Therefore, public awareness on the symptoms of tuberculosis and seeking early care is essential. Moreover, early detection, follow-up, and initiation of TB treatment among people living in rural areas and among the poor should be focused on.

## Abbreviations

CDR: Case detection rate
CI: Confidence interval
DOTs: Directly observed treatment short-course
ETB: Ethiopian birr
HBCs: High burden countries
HIV: Human immunodeficiency virus
HS: Health system
IQR: Inter quartile range
MDG: Millennium development goal
MDR-TB: Multi drug resistant tuberculosis
MTB: Mycobacterium tuberculosis
PTB+: Smear-positive pulmonary tuberculosis
SD: Standard deviation
SPSS: Statistical software for social science
TB: Tuberculosis
TTD: total treatment delay
WHO: World Health Organization
WRD: WHO-endorsed Rapid Diagnostics
